# Effect of “Tonifying Kidney and Invigorating Brain” acupuncture in children with spastic cerebral palsy analyzed by multi-modality MRI combined with dynamic electroencephalogram

**DOI:** 10.1097/MD.0000000000021082

**Published:** 2020-07-24

**Authors:** Dong Chen, Chao Bao, Yan-Xia Geng, Ming Yang, Elsie Sin May Teo, Jan-Bing Li, Yan-Cai Li, Nan Wang, Meng-Qian Yuan, Qin Zou, Ping-Ping Tang, Li-Li Zhu, Bin Xu

**Affiliations:** a Department of Acupuncture, Jiangsu Province Hospital of Traditional Chinese Medicine, Affiliated Hospital of Nanjing University of Traditional Chinese Medicine; b Nanjing University of Traditional Chinese Medicine; c Children^'^s Hospital of Nanjing Medical University, Nanjing, China.

**Keywords:** cerebral palsy, acupuncture, ambulatory electroencephalogram, multimodal magnetic resonance imaging

## Abstract

**Introduction:**

Cerebral palsy is the most common motor disability of childhood. Spastic cerebral palsy accounts for 60% to 70% of cases. Research has shown that acupuncture can improve the quality of life of children with cerebral palsy, but the mechanism of action remains unclear. This study aims to determine the effectiveness of acupuncture for treatment of children with spastic cerebral palsy and to assess the value of multimodal magnetic resonance imaging (MRI) and ambulatory electroencephalogram (EEG) for evaluation of treatment effect.

**Methods and analysis:**

This randomized controlled trial will enroll a total of 72 children with CP from 2 hospitals—Jiangsu Province Hospital of Chinese Medicine and Nanjing State Hospital of Pediatric—with 36 participants from each hospital. Patients will be randomly assigned (1:1 ratio) to receive “Tonifying Kidney and Invigorating Brain” acupuncture treatment plus standardized physical rehabilitation treatment (treatment group) or only standardized physical rehabilitation (control group). All participants will receive 3 treatment sessions per week for 3 consecutive months; they will then be followed up for another 3 months. The primary outcome measures will include multimodal magnetic resonance imaging (MRI), ambulatory electroencephalogram (EEG), and Gesell Developmental Diagnostic Schedules. The secondary outcome measures will include Gross Motor Function Classification System (GMFCS), Gross Motor Function Measure (GMFM), Functional Independence Measure (WeeFIM), and Modified Ashworth Scale score. Outcome measures (including primary and secondary outcome measures) were collected at the baseline, 3 months and 6 months prior to the intervention.

Ethics and dissemination

**Patients consent:**

Obtained.

**Ethics approval:**

The central independent ethics committee of Jiangsu Province Hospital of Traditional Chinese Medicine approved the protocol (2017NL-115-02).

**Safety considerations:**

Routine blood tests and liver and kidney function tests will be conducted to exclude patients with severe heart, liver, or kidney diseases. The same examinations will be performed again at the end of the study to detect any possible side effects. Possible acupuncture-related adverse events (e.g., fainting, needle stick injury, local infection, subcutaneous hematoma, and low-grade fever) will be documented. Serious adverse events will be reported to the principal investigator immediately. All unexpected and unintended responses, even those not necessarily related to the acupuncture intervention, will be documented as adverse events.

**Case dropout management:**

Participants have a right to withdraw from the study at any time if they feel uncomfortable upon receiving the treatments or being diagnosed with serious complications or diseases. They will then be referred to the preferred department for further treatment and management. If cases of dropout, the researcher need to contact the participant to reason the problem out, collect and record all the necessary assessments on the last visit as well as the date of last visit. All data available until the date of withdrawal will be stored for further statistical analysis.

**Discussion:**

This research is being conducted to assess the value of acupuncture as an intervention for rehabilitation of children with spastic cerebral palsy and also to evaluate the usefulness of multimodal MRI and ambulatory EEG for identifying changes in brain function.

**Trial registration:**

This trial is registered with Chinese Clinical Trials Register, ChiCTR 1900024546 (registered 15 July 2019; retrospective registration, http://www.chictr.org.cn/showproj.aspx?proj=35763).

**Strengths and limitations of this study**

- MRI and EEG can provide a more precise assessment of the treatment effect.
- The sample size of this study is not large.
- This study is confined to only 1 subtype of cerebral palsy, i.e., spastic cerebral palsy.
- MRI is most likely to be rejected by parents due to the prolonged duration of the examination.
- The results of MRI might vary or be unclear because children are unlikely to cooperate during the examination.

## 1 Introduction

Cerebral palsy (CP) comprises a group of permanent disorders of movement and posture caused by nonprogressive disturbances that occurred in the fetal or infant brain. The motor disorders of CP are often accompanied by disturbances of sensation, perception, cognition, communication, and behavior, and sometimes by epilepsy and secondary musculoskeletal problems.^[1]^ Currently, CP affects 1% to 5% infants worldwide, and the prevalence is reported to be increasing.^[2]^ In China, a survey of 6 provinces found a prevalence of 1.2% to 2.7%.^[3]^ According to 1 estimate there are a total of 400 to 500 million children with CP in China, 42% to 45% of whom are physically disabled, and around 3 to 4 million new cases are being added each year.^[4]^

Spastic CP, which is seen in 60% to 70% of cases,^[5]^ results from damage to areas of the brain that control reflex actions; affected children therefore present with increased muscle tone, decreased range of motion of the joints, disturbances of posture, and activity limitation. Treatment includes neurological rehabilitation (Bobath concept and Vojta method); basic rehabilitation (cerebral palsy rehabilitation and core stabilizing training); physical therapies (functional electrical stimulation, biofeedback therapy, and transcranial magnetic stimulation); assistive devices; occupational therapy; conductive education; surgery, psychological rehabilitation, and nursing care. While these treatments may reduce spasticity and improve motor function over the short term, the effects are not long lasting.

There is increasing interest in the use of traditional Chinese medicine, especially acupuncture, in CP, and acupuncture was even included in the 2015 China Guidelines Protocol in Cerebral Palsy Rehabilitation.^[6]^ Some studies have shown that acupuncture in combination with standardized rehabilitation can greatly improve motor function in children with CP and improve quality of life,^[7,8]^ but the mechanism of action, duration of efficacy, and individual differences in efficacy remains to be clarified.

This clinical trial aims to determine the effectiveness of acupuncture in spastic CP.

## 2 Methods

### 2.1 Study design

Randomized controlled trial comparing 2 treatments: “Tonifying Kidney and Invigorating Brain” acupuncture plus standard rehabilitation versus standard rehabilitation alone for treatment of children with spastic CP.

### 2.2 Setting

The study is currently underway in 2 different hospitals, Jiangsu Province Hospital of Chinese Medicine and Nanjing State Hospital of Pediatric.

### 2.3 Patient and Public Involvement

No patient involved.

### 2.4 Intended sample size

The following formula was used to calculate the intended sample size considering an average efficiency of 92% and 63% for the treatment and the control groups, respectively, α = 0.05, and β = 0.2: 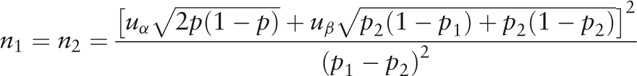


Thus, the estimated sample size for each group is 30. However, assuming a shedding rate of 20%, the sample size would increase by another 20%. Thus, the sample size intended for each group is 36, with a total sample size of 72.

### 2.5 Participants

**Figure 1:**
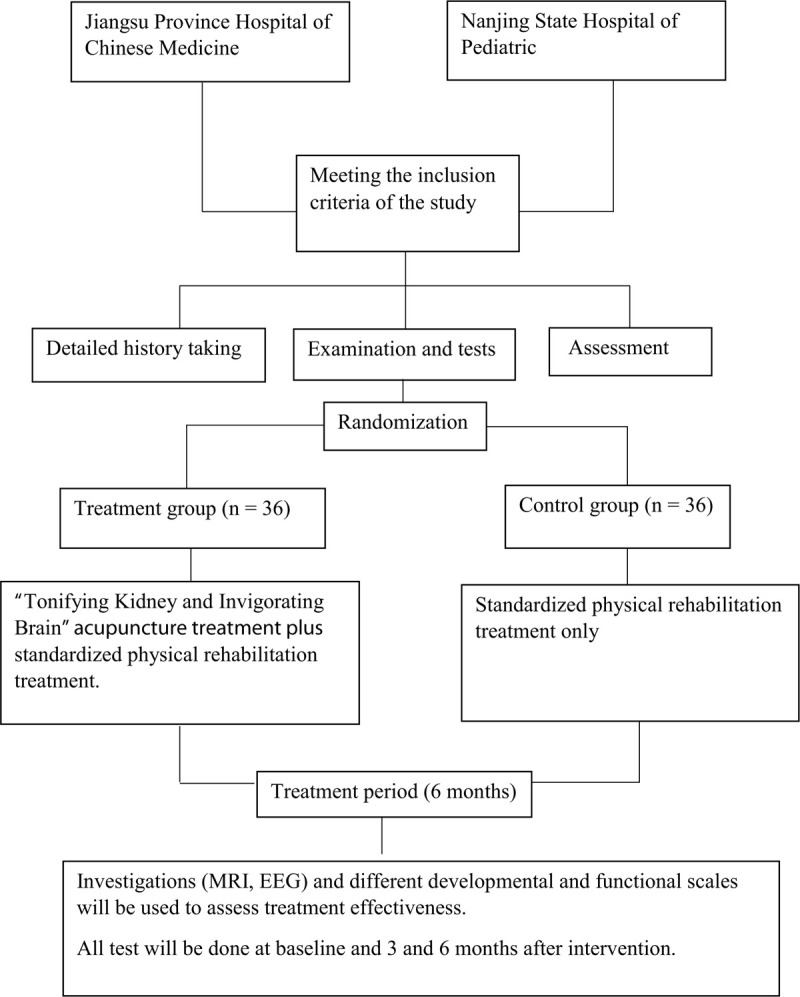
Participant flow diagram.

A total of 72 children with CP will be recruited (36 from each of the 2 hospitals) and randomly assigned to the treatment group or the control group in a ratio of 1:1 (Fig. 1).

Diagnosis will be according to 2015 China Guidelines Protocol in CP Rehabilitation:^[6]^

Main criteria:

1. Disorder of the central nervous system with persistent motor dysfunctionality
2. Abnormality of the development of movement and posture
3. Abnormality of muscle tone and muscle power
4. Abnormality in muscle reflex

Secondary criteria:

1. Evidence related to etiology of cerebral palsy.
2. Present of magnetic resonance imaging (MRI) report

According to the guidelines there are 6 types of CP: spastic quadriplegia, spastic diplegia, spastic hemiplegia, ataxic CP, mixed CP, and dyskinetic CP. Only children with spastic forms of CP will be considered for inclusion in this study.

*Inclusion criteria*:

1. diagnosis of spastic CP,
2. age between 6 months and 7 years,
3. willingness to participate in the study (parents sign the inform consent form on behalf of their children).

*Exclusion criteria*:

1. severe form of spastic CP or
2. presence of a serious comorbidity (e.g., congenital heart disease, hydrocephalus, increased intracranial pressure, psychiatric or psychological disorders, liver or kidney dysfunction, or chronic infectious disease).

### 2.6 Informed consent

Trial objectives, characteristics, probable benefits and risks, alternative treatments available, and the participants rights and obligations, as stated in the Declaration of Helsinki, will be explained to the parents of the patients before obtaining their written consent. A representative from the research team approached families and described the study. Families were given adequate time to reflect on the information, had any questions answered and gave free and voluntary consent. Parents or legal guardians then provided written informed consent. If new ethical issues emerge during the course of the trial, the informed consent format will be revised and resubmitted to the Ethics Committee for approval; then, the participants consent will be requested again. In case any patient withdraws from the study, all data available till the date of withdrawal will be included in the final analysis.

### 2.7 Confidentiality

Participants information and medical record will be kept in a locked file cabinet in the personal possession of the researcher. Only the researcher and the members of the researchers committee will have the rights to access and review the medical data. Information from this research will be used solely for the purpose of this study and any publications that may result from this study. All participants involved in this study will not be identified and their anonymity will be maintained.

### 2.8 Recruitment

There will be 2 primary strategies in recruiting participants with CP. The first strategy is to recruit participants who are patients in the Department of Acupuncture in Jiangsu Province Hospital of TCM and Children^'^s Hospital of Nanjing Medical University. Second, we will post on-line advertisements to briefly introduce our study and recruit patients who are willing to participate.

### 2.9 Randomization

Enrolled patients will be randomized into the treatment (acupuncture plus standardized rehabilitation) group or the control (standardized rehabilitation alone) group in a ratio of 1:1, using the “sample” function in R (https://cran.r-project.org/). The randomization code will be placed inside sealed and opaque envelopes that will be opened only after recruitment of the patients.

Criteria of dropout:

1. Participant failed to continue the follow-up due to personal reason or unable to cooperate fully during the assessments.
2. Being diagnosed with serious complications or diseases.
3. Parents/guardian self-requested for termination of the treatment.

### 2.10 Case dropout management:

Participants have a right to withdraw from the study at any time if they feel uncomfortable upon receiving the treatments or being diagnosed with serious complications or diseases. They will then be referred to the preferred department for further treatment and management. If cases of dropout, the researcher need to contact the participant to reason the problem out, collect, and record all the necessary assessments on the last visit as well as the date of last visit. All data available until the date of withdrawal will be stored for further statistical analysis.

### 2.11 Safety monitoring

Before randomization, blood will be drawn for routine blood tests and liver and kidney function tests so that patients with severe heart, liver, or kidney diseases can be identified and excluded. The same examinations will be performed again at the end of the study to look for possible side effects (Table 1). Possible acupuncture-related adverse events (e.g., fainting, needle stick injury, local infection, subcutaneous hematoma, and low-grade fever) will be documented. Serious adverse events will be reported to the principal investigator immediately. All unexpected and unintended responses, even those not necessarily related to the acupuncture intervention, will be documented as adverse events.

**Table 1:**
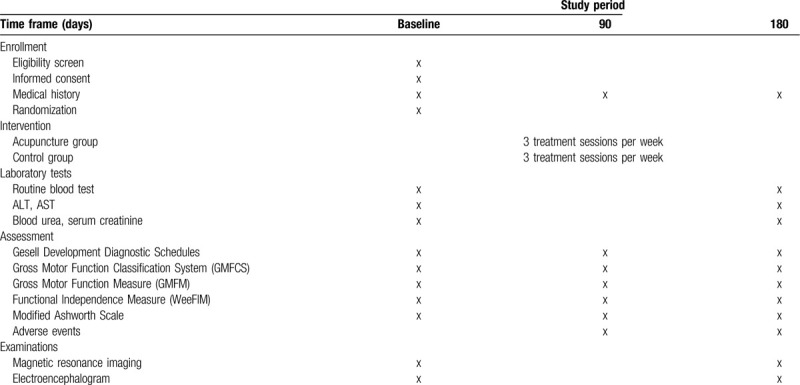
Schedule for enrollment, intervention, and assessments.

### 2.12 Interventions

All participants will receive 3 treatment sessions per week for 3 consecutive months. The treatment group will receive standardized rehabilitation therapy plus acupuncture; the control group will receive only standardized rehabilitation therapy (Fig. 1).

#### 2.12.1 Acupuncture

The acupuncture points will be identified according to the method recommended by the World Health Organization. The area will be sterilized before puncture. Acupuncture needles (25 mm in length and 0.30 mm in diameter) manufactured by Suzhou Huato Medical Instruments Co. Ltd., Suzhou, China, will be used for all patients.

The following acupoints will be used:

1. Local acupoint: Da Zhui (GV14), Sheng Zhu (GV12), Ming Men (GV4), Shen Shu (BL23), Zu San Li (ST36), San Yin Jiao (SP6), and Tai Xi (KI3). The needle will be inserted and removed immediately after manipulation of needle (twisting and rotating manipulation, with neutralizing technique between enforcing and reducing for 2 seconds) is done.
2. Scalp acupoints: Si Shen Zhen (1.5 cun superior, inferior, lateral, and medial to GV20; 4 needles in total); Zhi San Zhen (intersection of frontal hairline section with midline of the head, with 3 cun lateral and medial to the midline point; 3 needles in total); Nao San Zhen (1.5 cun lateral and medial to GV17; 3 needles in total); and Nie San Zhen (2 cun above the tip of the ear as the first acupoint, with 1 cun lateral and medial to the current point, on bilateral sides of the head; 6 needles in total). Si Shen Zhen will be needled obliquely 0.8 to 1 cun with the tip of the needle pointing away from GV20; Zhi San Zhen will be needled obliquely 0.5 to 0.8 cun; and Nie San Zhen and Nao San Zhen will be needled obliquely 1 cun inferiorly. Needle insertion will be at an angle of 20° to 30°. No lifting/thrusting manipulation will be done after needle insertion; the needle will be left in situ for 30 minutes. Electrical stimulation will be applied only in patients without history of seizure/epilepsy. The scalp acupoint will be connected to G-6805, an electrical acupuncture stimulation device. A pair of electrodes will be connected to the acupoints on both sides of the scalp, where Si Shen Zhen (the acupoint located lateral or medial to GV20) is connected to Nie San Zhen (the acupoint located directly 2 cun above the tip of the ear) on both sides of the scalp, and continuous waves stimulation will be applied for 30 minutes.

#### 2.12.2 Standardized physical rehabilitation

Standardized physical therapy will include comprehensive physical rehabilitation and biofeedback therapy. Patients will attend 3 30-minute rehabilitation sessions per week for a total of 3 months. The physical rehabilitation sessions will be conducted one-to-one in a quiet and safe environment so that the child is relaxed. Parents will be advised to bring the child for treatment in the mornings when their ability to concentrate on tasks will be maximum.

### 2.13 Outcome assessment

Outcome will be assessed at baseline, 3 and 6 months after the start of treatment using the modalities and instruments described below. (Table 1)

#### 2.13.1 Primary outcome measures

##### 2.13.1.1 Multimodal magnetic resonance imaging (MRI)

Multimodal MRI can provide functional and structural information. The comprehensive assessment might contribute to the development of new perspective in terms of the diagnosis, treatment principle, assessment as well as the prognosis of the disease.

##### 2.13.1.2 Ambulatory electroencephalogram (EEG)

Conventional EEG directly reflects the functionality of the brain activity,^[9,10]^ but its value is limited in the pediatric age-group, especially in children with CP, as they are unlikely to cooperate during the examination. Ambulatory EEG can record brain waves in various states (e.g., during sleep, physical activity).

##### 2.13.1.3 Gesell Development Diagnostic Schedules (GDDS)

The GDDS is widely used in medical research for assessing intellectual development of children aged 0-6 years. It can be used for children with disabilities.

#### 2.13.2 Secondary outcome measures

##### 2.13.2.1 Gross Motor Function Classification System (GMFCS):

GMFCS reflects the gross motor function of children with CP. It classifies children with CP into 5 different age-groups, with 5 levels in each age-group, according to the childs current motor function. It is a measure of the severity of the childs motor dysfunction.

##### 2.13.2.2 Gross Motor Function Measure (GMFM):

The GMFM is widely used to measure changes in gross motor function of children with CP. It assesses 5 different dimensions of the childs motor function: A) lying and rolling (17 items); B) sitting (20 items); C) crawling and kneeling (14 items); D) standing (13 items); and E) walking, running, and jumping (24 items). Each gross motor skill is scored to note its presence or absence (or emergence). Each item is scored on a scale of 0 to 3 (0 = unable, 1 = attempted, 2 = partially accomplished, 3 = completed).

Calculation of dimension percentage scores:

**Figure:**
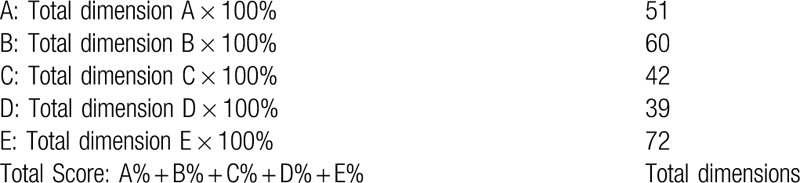


Goal Total Score: Sum of % scores for each dimension identified as a goal area Number of Goal Areas

##### 2.13.2.3 Functional Independence Measure (WeeFIM):

WeeFIM measures a childs consistency in the performance of essential daily functions. Three main domains (selfcare, mobility, and cognition) are assessed by interviewing or by observing a childs performance of a task to criterion standards. WeeFIM is categorized into 2 main functional streams: “Dependent” and “Independent”. The total score can range from 18 to 126 (mobility: 91, cognition: 35), with 126 = completely independent, 108–125 = basically independent, 90–107 = mildly dependent, 72–89 = mildly dependent, 54-71 moderately dependent, 36–53 = severely dependent, 19–35 = most severely dependent, and 18 = completely dependent.

### 2.14 Statistical methods

Normally distributed data will be summarized as means (± standard deviation) and non-normally distributed data as medians (range). The Student t test or the Wilcoxon rank sum test will be used to analyze differences between the groups. If there is heterogeneity of variance, then Satterthwaite corrected *t* test will be used. *P* ≤ .05 will be considered statistically significant and *P* ≤ .0.01 will be considered highly significant.

## 3 Discussion

Neuroplasticity or the ability of the brain to modify its structure and function in response to changes in the body or in the external environment^[11]^ is one of the mechanisms whereby motor function recovery following brain injury occurs. One study has also indicated that plasticity of the brain can be boosted by improving the motor function of the trunk and limbs.^[12]^ Neuroplasticity and compensatory capacity is highest during childhood^[13–15]^ and so early intervention in children with CP is recommended. Acupuncture has been used widely in children for managing various neurological disorders, including CP. Acupuncture has shown much greater efficacy than other therapies in several studies, and parents find it acceptable because of the minimal side effects.^[16–19]^ However, although the overall response rate with acupuncture is in the range of 61% to 100%, still there no clarity regarding its mechanism of action in children with CP. This study therefore aims to throw some light on the mechanism of action of acupuncture in CP. We will also examine the use of multimodal MRI and ambulatory EEG for objective assessment of any pathological and functional changes. The Radiology Department of Nanjing State Hospital of Pediatric will be interpreting the multimodal MRI and the ambulatory EEG recordings obtained during the course of this study.

In conclusion, this research aims to provide evidence that the combination of “Tonifying Kidney and Invigorating Brain” acupuncture and standard rehabilitation can improve brain function of children with CP. We also try to determine the usefulness of multimodal MRI and ambulatory ECG for evaluation of acupuncture treatment.

## Acknowledgments

We acknowledge Rehabilitation Department and Radiology Department of Nanjing Children's Hospital for recruiting children with cerebral palsy. We also appreciate the help and efforts of all research staff participating in this trial.

## Author contributions

Authorship: Dong Chen, Chao Bao, Yan Xia Geng, Ming Yang, and Bin Xu conceived this trial and participated in the design of the trial. Elsie Sin May Teo revised the manuscript. Yan Cai Li, Nan Wang, Qin Zou, Ping Ping Tang, and Li Li Zhu monitored the study. All authors read this manuscript and approved the publication of this protocol.

**Conceptualization:** Dong Chen, Chao Bao, Ming Yang, Bin Xu.

**Project administration:** Bin Xu.

**Review and editing of the manuscript:** Meng Qian Yuan, Elsie Sin May Teo.

**Supervision:** Yan Xia Geng, Jan Bing Li.

**Validation:** Yan Cai Li, Nan Wang.

**Visualization:** Qin Zou, Ping Ping Tang.

**Writing original draft:** Li Li Zhu.
